# Stability Bounds for Micron Scale Ag Conductor Lines Produced by Electrohydrodynamic Inkjet Printing

**DOI:** 10.1021/acsami.2c11133

**Published:** 2022-08-18

**Authors:** Jinxin Yang, Pei He, Brian Derby

**Affiliations:** † Department of Materials, University of Manchester, Oxford Rd., ManchesterM13 9PL, U.K.; ‡ School of Physics and Electronics, Hunan Key Laboratory of Nanophotonics and Devices, Central South University, Changsha, Hunan410083, P. R. China

**Keywords:** electrohydrodynamic jetting, inkjet, printing, printed electronics, contact angle

## Abstract

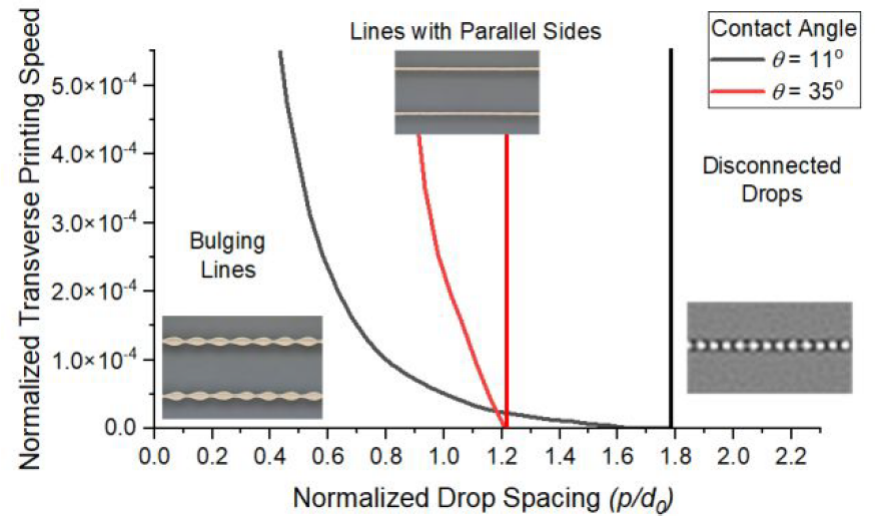

Continuous conducting lines of width 5–20 μm have been printed with a Ag nanoparticle ink using drop-on-demand (DOD) electrohydrodynamic (EHD) inkjet printing on Si and PDMS substrates, with advancing contact angles of 11° and 35°, respectively, and a zero receding contact angle. It is only possible to achieve stable parallel sided lines within a limited range of drop spacings, and this limiting range for stable line printing decreases as the contact angle of the ink on the substrate increases. The upper bound drop spacing for stable line formation is determined by a minimum drop overlap required to prevent contact line retraction, and the lower bound is governed by competing flows for drop spreading onto an unwetted substrate and a return flow driven by a Laplace pressure difference between the newly deposited drops and the fluid some distance from the growing tip. The upper and lower bounds are shown to be consistent with those predicted using existing models for the stability of inkjet printed lines produced using piezoelectric droplet generators. A comparison with literature data for EHD printed lines finds that these limiting bounds apply with printed line widths as small as 200 nm using subfemtoliter drop volumes. When a fine grid pattern is printed, local differences in Laplace pressure lead to the line width retracting to the minimum stable width and excess ink being transported to the nodes of the grid. After printing and sintering, the printed tracks have a conductivity of about 15%–20% of bulk Ag on the Si substrate, which correlates with a porosity of about 60%.

## 1 Introduction

Inkjet printing has been used as a manufacturing tool for a wide range of applications beyond its original use for text and graphics printing.^1−4^ Conventional inkjet printing forms drops of fluid, either through the Rayleigh instability of a liquid stream (continuous inkjet printing - CIJ) or the pinch-off of a single drop from an ejected column or jet of liquid driven by the minimization of surface energy (drop on demand inkjet printing - DOD). Of these two drop formation mechanisms, DOD is more commonly used for nongraphics applications because it is compatible with a greater range of inks. However, the physical limitations of conventional drop generators, in particular the need to overcome the Laplace pressure at small drop sizes and the resistance to fluid flow at small tube diameters, make it difficult to achieve droplets with size smaller than about 1 pL.^5^ This limits its spatial resolution on a substrate to >20 μm. With the increasing demand of patterning on surfaces at micrometer and submicrometer length scales for electronic applications, electrohydrodynamic (EHD) inkjet printing has been developed to achieve direct high resolution printing by droplet deposition with drop volumes in the fL or smaller sized range. Here we investigate the factors that limit the stability of fine conducting lines at the micron scale produced by EHD droplet deposition.

It has been known for some time that the action of electrostatic forces on the exposed surface of a confined fluid cylinder can force the surface into a conical shape (the Taylor cone), and under appropriate conditions, the electric field results in the ejection of droplets.^6^ The Taylor cone and the small scale of nozzle diameter allow EHD inkjet printing with ejected droplets in the femtoliter volume, thus enabling direct ultrahigh resolution patterning in arbitrary geometries.^7^ With the advantages of high resolution printing and its compatibility with a wide range of inks (1–10,000 mPa s),^8^ EHD inkjet printing has been used to deposit a variety of inks, including metal nanoparticle suspensions,^9,10^ nanowires,^11,12^ polymers,^13,14^ and biomaterials.^15−17^

To achieve stable DOD printing, the pulsating mode and pulsed cone jet mode are applied. The pulsating mode uses the intrinsic natural frequency of the jetting mode to generate droplets. Pulsating jets occur due to the imbalance between the supply and loss of fluid, and the natural frequency strongly depends on the flow rate and electric field, *E*.^18^ Chen et al.^19^ proposed that for pulsating EHD printing, the flow rate is controlled by a balance between electric stress, applied pressure, and capillary pressure. They used dimensional analysis to propose that the pulsating frequency, *f*, scales with *E*(2) corresponding to a high-frequency mode. However, the limitation of using the intrinsic pulsating frequency to achieve drop on demand EHD printing is also obvious. The ejected droplet diameter and jetting frequency are both influenced by the electric field; thus, it is impossible to change one parameter and maintain the other by adjusting the applied voltage, which limits the control of sizes of deposited droplets.

Pulsed cone jet EHD printing can achieve DOD deposition with controllable jetting frequency and droplet size by the use of a pulsed electric field.^20−23^ This leads to periodic formation and relaxation of the Taylor cone to eject droplets with a pulsed electric field; thus, the control of the pulse voltage and duration is important to achieve DOD beyond intrinsic jetting.^8^ Park et al. achieved DOD patterning by using pulsed EHD printing and developed a model to predict the droplet size and resulting printed line width by combining the scaling law for EHD printing and the volume conservation method used to estimate the line width with conventional DOD printing systems.^24^ A similar model to predict feature size for both pulsed and continuous EHD printing was developed by Qian et al.^25^ However, there has been little systematic study of the stability of deposited features under EHD inkjet printing conditions.

It is well-known that when a linear feature is formed through the sequential deposition of drops, the stability of the resulting line is controlled through a combination of static and dynamic equilibria. Davis demonstrated that a linear liquid feature is stable if there is significant hysteresis between the advancing and receding contact angles.^26^ In which case, volume conservation leads to a simple relation with the line width controlled by the volume and spacing of drops along with the contact angle. Both Soltman and Subramanian^27^ and Derby and Stringer^28−30^ observed that there was only a limited range of drop spacings that resulted in stable parallel sided lines as required for printed conductors, with a wavy line forming if the drop spacing was too large and, if the drops were too closely spaced, irregular bulges occurring on an otherwise well-formed line. To the best of our knowledge, there has been no systematic study of the morphology of lines produced by the coalescence of drops deposited by EHD DOD printing. A brief review of the literature reveals that EHD printing has been used to fabricate continuous parallel lines with a range of different material inks, including suspensions of Ag nanoparticles,^20,23,24,31−35^ Au nanoparticles,^36^ Indium–Tin Oxide (ITO) nanoparticles,^37^ Cu nanoparticles,^38,39^ graphene,^40^ and polymer solutions,^41^ with line widths ranging from about 100 nm^36^ to >100 μm.^37^ In a number of cases, transitions in behavior occur from coalesced drops to stable lines, followed by unstable bulged lines, as reported earlier for conventional DOD inkjet printed lines. Full details of the ink fluid properties and printing conditions reported in these studies are presented in the Supporting Information, Table S1.

The primary objective of this study is to study the limited range of drop spacing that can be used with EHD DOD printing to produce lines with parallel sides. In particular, we will test the validity of equilibrium and dynamic models developed earlier to explain these phenomena when lines are printed with conventional inkjet printers,^29^ which has been validated for drops with volumes >10 pL.^30^ The predictions of the model will also be tested using literature data on EHD DOD printing to assess its applicability with decreasing drop volume.

## 2 Materials and Methods

The ink used in the experiments was a commercial silver nanoparticle ink (Silver dispersion, 736465, Sigma-Aldrich, Gillingham, UK) with density 1450 kg m^–3^ (manufacturer’s data). The viscosity of the ink was 6.6 mPa·s, measured using a Discovery HR-3 hybrid rheometer (TA Instruments, New Castle, DE, USA) at a shear rate of 1000 s^–1^. The surface tension was 28.0 mN·m^–1^, measured using a drop shape analyzer (DSA 100, Krüss, Hamburg, Germany). The Ag nanoparticle suspensions were deposited onto two substrates: 1) a Si wafer (n-type, 647799, Sigma-Aldrich, Gillingham, UK) and 2) a polydimethylsiloxane (PDMS) (101697, Onecall, Leeds, UK) coated Si wafer; the PDMS was applied to the silicon by spin coating (Ossila, Solpro Business Park, Sheffield, UK) at 2000 rpm for 1 min followed by curing at 70 °C for 2 h in an oven (Genlab, MINO/30/F/PDIG, Widnes, UK). The resulting PDMS layer had a thickness of approximately 50 μm. Prior to printing, the Si and PDMS substrates were cleaned using a UV-ozone surface treatment (ProCleaner, Bioforce Nanosciences, USA) for 10 and 40 min, respectively. The advancing and receding contact angles of each ink/substrate combination were measured using the DSA 100, and the contact angle conditions on each substrate are shown in Table 1.

**Table 1:** Advancing and Receding Contact Angles of the Ink on the Two Substrates Used in This Study

substrate	adv. contact angle	rec. contact angle
silicon	11° ± 1°	0°
PDMS	35° ± 1.5°	0°

**Figure 1:**
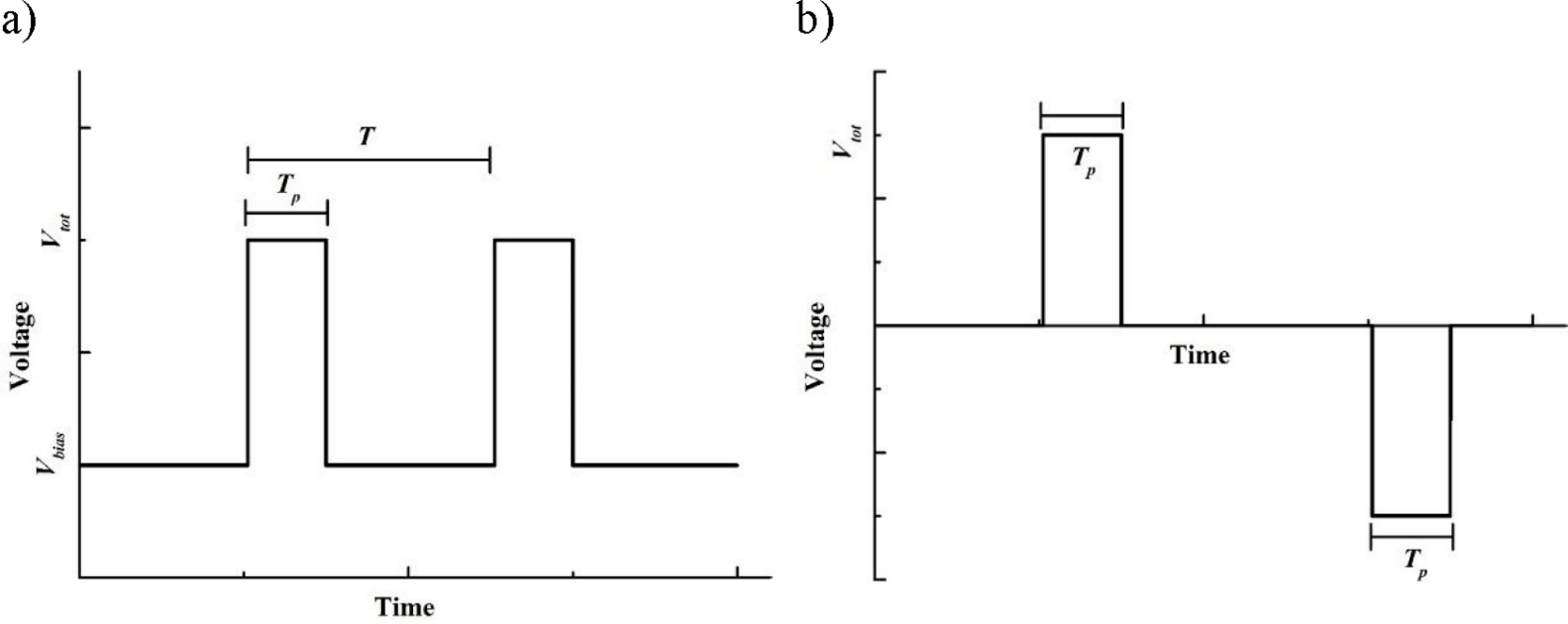
Waveform used in the EHD inkjet printing experiments: a) a single pulse waveform (Si substrate) and b) a bipolar waveform (PDMS substrate). *V*_*bias*_ is the bias voltage, *V*_*tot*_ is the total applied voltage, and *T*_*p*_ is the pulse width time.

The printer used in the experiments is a commercial EHD printing system (SLTS0505-KBD, SIJ Technology, Tsukuba, Japan), equipped with a Superfine nozzle of a 1.8 μm internal diameter. Full details of the methods used to determine the optimum printing conditions are given in the Supporting Information. The printing parameters used are as follows. The printing nozzle tip is fixed 20 μm above the substrate. Printing on the Si substrate was achieved by superimposing a pulsed voltage of 210 V above a fixed bias voltage of 100 V (total 310 V). The PDMS substrate is electrically insulating; hence to prevent charge accumulation, a bipolar waveform was used with alternating positive and negative pulses, as proposed by Son et al.^31^ In this case, the pulse voltage used was ±250 V. The actuating waveforms used on both substrates are illustrated in Figure 1.

For pulsed EHD inkjet printing, the volume of the drop ejected per pulse, *V*, is determined by the mean fluid flow rate through the printer nozzle, *Q*, and the pulse duration, *T_p_*, with
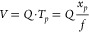
1where *x*_*p*_ = *T*_*p*_/*T* is the duty ratio of the pulse, and *f* = 1/*T* is the actuating pulse frequency. Thus, if the droplet deposition frequency is changed, in order to maintain a constant drop volume the actuating pulse width must remain constant, and the pulse duty ratio must be changed. The influence of the actuating waveform on the drop volume is illustrated in the accompanying Supporting Information (Figures S2 and S3). This effect is important if the drop spacing and printer transverse velocity (printing speed) are to be studied while maintaining a constant drop volume. Drop volume was calculated by printing isolated drops on the desired substrate, measuring the diameter of the resulting equilibrium sessile drop, and using the advancing contact angle to calculate the drop volume assuming the geometry of a spherical cap (Supporting Information Figure S4). For printing on the Si substrates, a constant drop volume of 11 fL was used (equivalent drop diameter in flight, *d*_*o*_*=* 2.8 μm). Using the bipolar actuation of the PDMS substrate led to a slightly larger drop volume of 17 fL, with *d*_*o*_ = 3.2 μm. In order to study the influence of drop spacing, *p*, and printing velocity, *v*, on line width and stability, drop deposition frequency and drop spacing were adjusted using the relation *v = pf*. The EHD printer had a maximum operating frequency of 1 kHz, and this allowed printing speeds in the range of 0.1–2.0 mm s^–1^ with the drop spacing ranging from 1–7 μm. The complete printer settings used to obtain each drop spacing and velocity combination are provided in the Supporting Information.

To investigate the electrical properties of the printed silver lines, we used the following test structure. A 2 mm length line was printed using a pattern of overlapping single drops between two 100 μm × 100 μm squares of printed Ag acting as contact electrodes. The line was overprinted, if required up to a maximum of 15 printed layers. After printing, the deposited structures were annealed at 150 °C for 1 h in air in a laboratory oven (Genlab, MINO/30/F/PDIG, Widnes, UK). The conductance of the printed line was measured using a two-point probe (Jandel Engineering, Linslade, UK) between the square electrodes, coupled to a voltmeter and current source (Keithley 2400, Cleveland, OH, USA).

## 3 Results and Discussion

### 3.1 Printed Line Morphology

**Figure 2:**
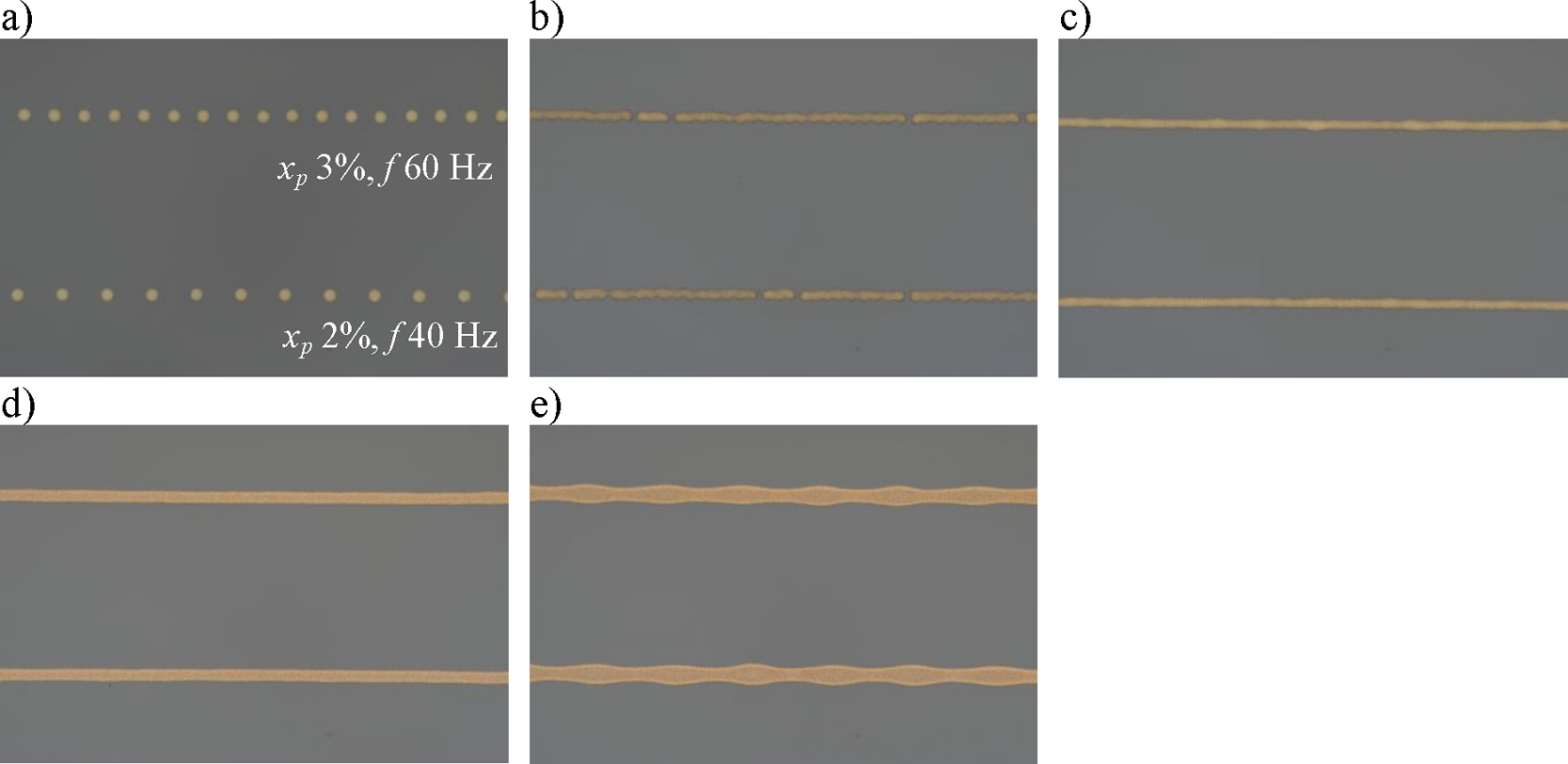
Different line morphologies observed: a) individual droplets: printing speed, *U*_*T*_ = 1 mm s^–1^, drop spacing, *p* ≫ drop diameter; b) short discontinuous lines: *U*_*T*_ = 1.96 mm s^–1^, *p* = 7 μm; c) continuous lines with irregular edges: *U*_*T*_ = 1.92 mm s^–1^, *p* = 6 μm; d) continuous parallel sided lines: *U*_*T*_ = 1 mm s^–1^, *p* = 4 μm; e) bulging lines: *U*_*T*_ = 0.2 mm s^–1^, *p* = 2.5 μm.

To investigate the stability of printed line structures formed with femtoliter drops, a series of prints were carried out on the Si and PDMS substrates, with printing speed ranging from 0.1 to 2 mm s^–1^ and drop spacing ranging from 1 to 7 μm. The frequency and duty ratio combinations are listed in the Supporting Information Tables S2 to S8. The printer control system allows only integer values for the pulse and cycle duration; hence, some printing velocity and drop spacing combinations cannot be achieved, and an approximate printing velocity was recorded in these cases, as listed in the Supporting Information. The resulting printed structures show five distinct morphologies depending on the drop spacing and printing speed (Figure 2 and Table 2). A complete set of images of lines printed under all conditions studied is presented in the Supporting Information Figures S5 and S6. These distinct line structure morphologies are identical to those reported in previous work on the printing of lines by droplet deposition using conventional inkjet printing.^27,29,30^ Note that the conditions for stable line deposition are valid over a much smaller range of droplet spacing on the PDMS substrate than with the Si substrate.

**Table 2:**
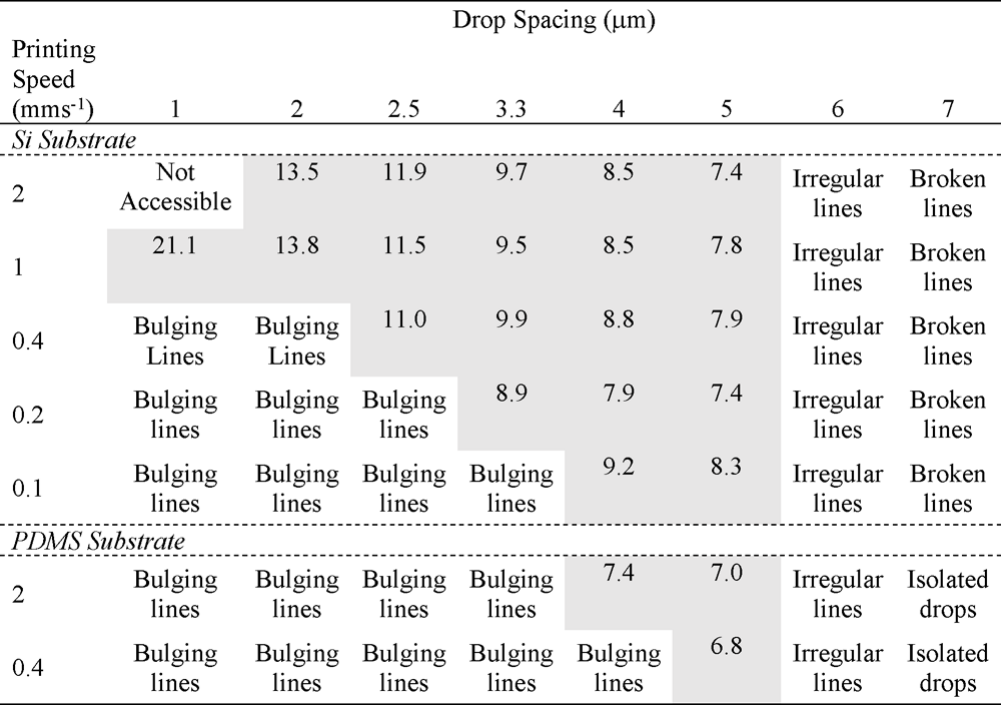
Printed Line Morphology at Each Set of Printing Conditions on the Two Substrates^a^

a Where regular lines with parallel edges form (shaded), the line width is given in μm.

**Figure 3:**
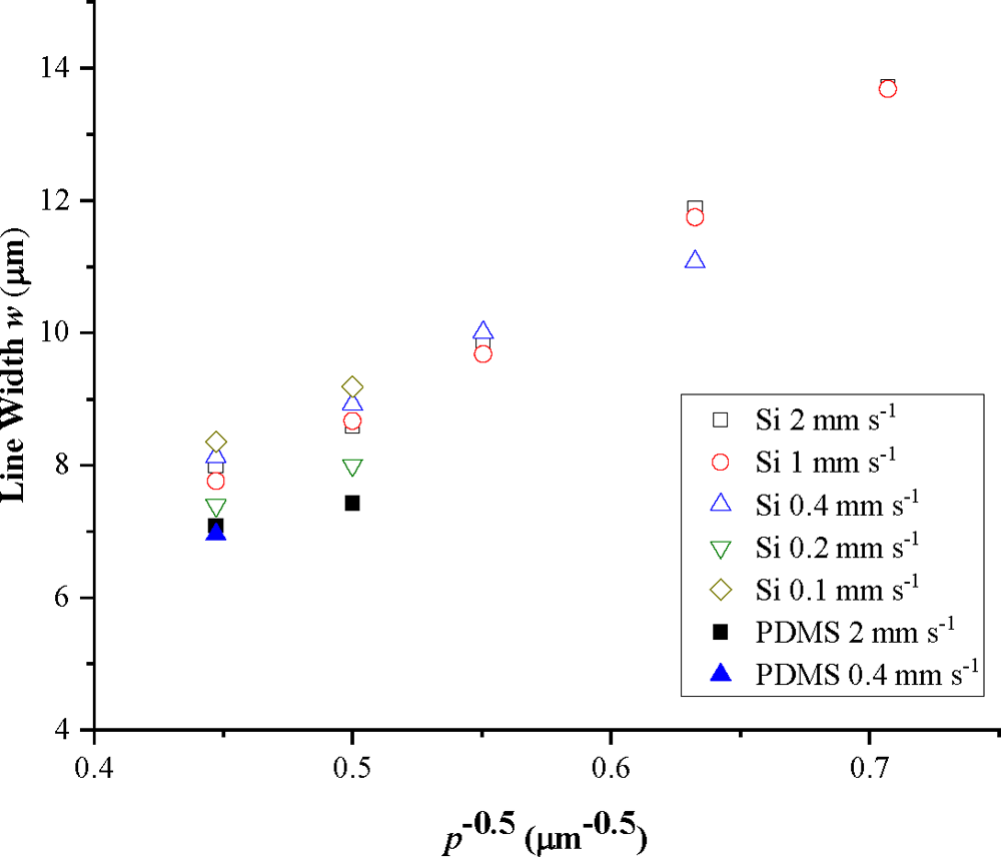
Width of a parallel sided printed line plot as a function of the drop spacing raised to the power of −0.5, following eq 2. The data from the Si substrate shows the expected linear relation. There is insufficient data from the PDMS substrate to confirm this prediction, but the data is broadly consistent with the expected drop volume.

For the case of continuous parallel sided lines, the width of the line, *w*, can be predicted assuming conservation of volume with overlapping drops separated by a distance, *p*, and an equilibrium contact angle, θ, using the following relation proposed by Smith et al.^28^
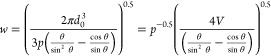
2where *d*_*o*_ is the diameter of the spherical drop in flight prior to impact. Stringer proposed that the line width from eq 2 showed better agreement with θ replaced by the advancing contact angle (*θ*_*a*_).^29^ Thus, by plotting the width of the line as a function of *p*^–0.5^, the gradient will be a simple function of the advancing contact angle and drop volume, *V* (Figure 3). Using this data the mean drop volume deposited onto the Si substrate is calculated as 10.3 fL, which is slightly less than the value of 11 fL determined from measurements of isolated sessile drops. There are only three conditions under which parallel lines could be printed using the PDMS substrates, and analysis of this data results in a mean drop volume of 25 fL, which is larger than that determined from isolated drops (17 fL). The values for the drop volume and equivalent drop diameter obtained from the line width data are used in subsequent discussions of the printed lines.

### 3.2 Limiting Bounds for Parallel Sided Lines

Table 2 shows the minimum and maximum drop spacing between which parallel lines form. The spacing, above which the line begins to transition to isolated drops, is common to both substrates and independent of printing speed. However, the minimum spacing, below which bulges appear, is clearly a function of both the printing speed and the advancing contact angle, such that the range of drop spacing where a stable line forms reduces as the printing speed decreases and as the contact angle increases. Stringer and Derby proposed that the maximum drop spacing is controlled by the diameter of the equilibrium sessile drop
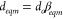
3with
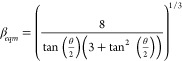
4where *d*_*o*_ is the diameter of the drop in flight. In the case of a zero receding contact angle, *d*_*eqm*_ defines a minimum line width at which a parallel sided liquid ridge can form without contact line retraction.^29^ Assuming volume conservation, eqs 3 and 4 can be combined with eq 2 to define the maximum drop spacing for printing parallel sided lines.
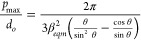
5

To predict the minimum drop spacing, below which the bulging instability occurs, Stringer used a model developed by Duineveld,^42^ which considered a balance between the fluid flow forward associated with a fresh drop arriving on the surface and a flow back along the printed line driven by the Laplace pressure difference between the new drop and that in the equilibrium liquid line. This dynamic model includes the printing speed or traversing velocity, *U*_*T*_. This leads to the following inequality relating the printing speed and minimum drop spacing at which stable lines can be printed without the formation of bulges
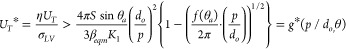
6with
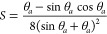
7and
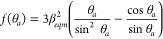
8Here *U*_T_* is the normalized printing speed; *U*_*T*_ is normalized by ink viscosity, η, and ink surface tension, *σ*_*LV*_; θ_*a*_ is the advancing contact angle; and *K*_1_ is a constant close to 1. Note that *U*_T_* has the same dimensionless form as the capillary number but should not be confused with it.

**Figure 4:**
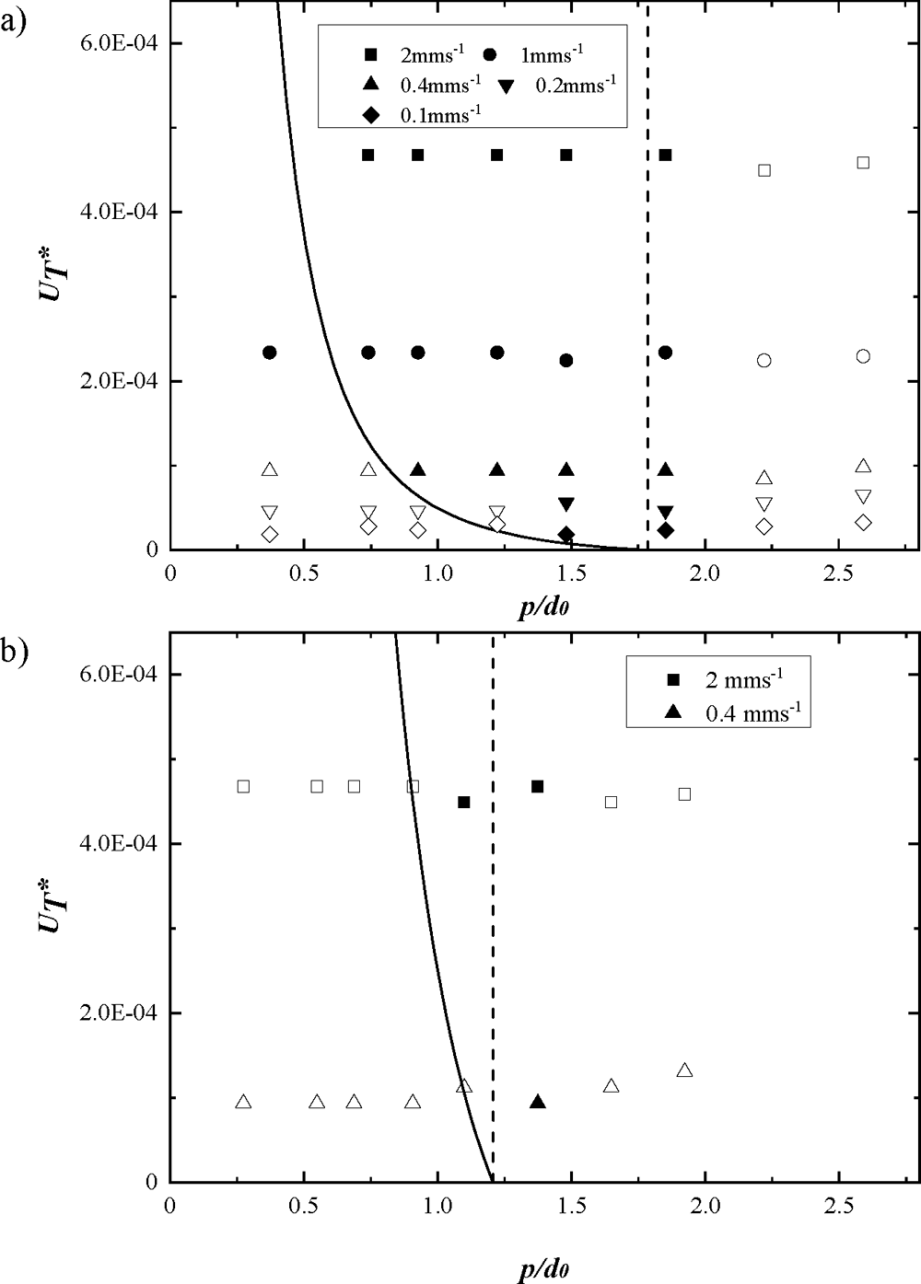
Stability map showing the condition for the maximum (dashed line) and minimum (solid line) drop spacings, normalized by the diameter of a drop in flight. Between these limits, stable parallel sided lines can be printed at a given normalized printing speed on the substrates used in this study: a) Si substrate and b) PDMS substrate. Filled symbols indicate where stable parallel sided lines were observed in the experiments.

Eqs 5 and 6 predict the maximum and minimum drop spacings at a given printing speed for any given ink/substrate combination. These are presented in Figure 4 using a normalized drop spacing, with p* = p/d, and compared with experimental results from the Si and PDMS substrates. The experimental results show good agreement with the predictions. In all cases, the maximum allowable drop spacing (dashed line in Figure 4) is predicted to be independent of the printing speed and controlled by the contact angle. However, the minimum allowable drop spacing is a function of the printing speed, with a larger range of available drop spacing as the printing speed increases. The stability range is also a function of the ink/substrate contact angle with the larger contact angle found with the PDMS substrate leading to a significantly smaller range of stable line printing.

**Figure 5:**
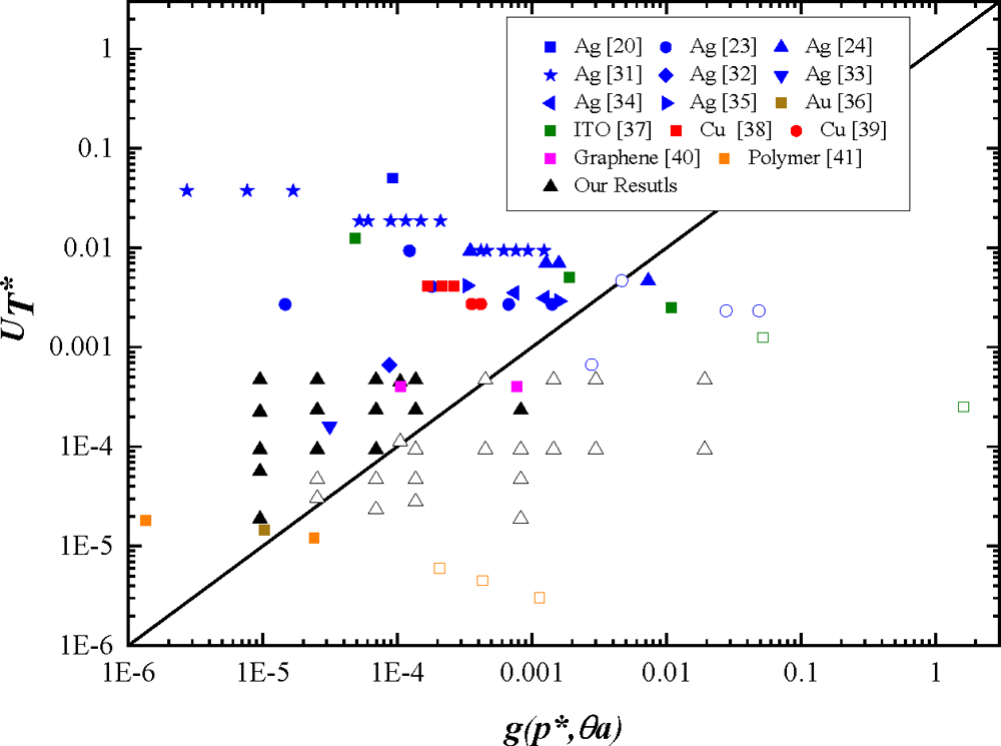
Predicted value for the minimum drop spacing as determined by the inequality in eq 6 (solid line). Experimental data from this study (black symbols) and the literature (colorful symbols), including Ag nanoparticles,^20,23,24,31−35^ Au nanoparticles,^36^ ITO nanoparticles,^37^ Cu nanoparticles,^38,39^ graphene,^40^ and polymer^41^ inks, are superimposed. Filled symbols represent stable parallel sided lines successfully printed, while open symbols indicate the onset of a bulging instability.

The predictions of the model can also be compared with experimental data published in the literature for the conditions under which stable lines have been printed using EHD inkjet printing. In order to compare data from a range of publications that have used EHD printing for a range of inks and substrates, it is more convenient to focus on the bulging instability and plot the inequality in terms of the dimensionless velocity *U*_T_* and the function g*(p/d_*o*_,*q)*, which captures both the influence of the drop spacing and contact angle. This is shown in Figure 5 with the data for the work in this study and that published in earlier studies from the literature with a wide range of inks including Ag nanoparticles,^20,23,24,31−35^ Au nanoparticles,^36^ ITO nanoparticles,^37^ Cu nanoparticles,^38,39^ graphene,^40^ and block copolymer (hydroxyl-terminated random copolymers in 1,2,4-trichlorobenzene).^41^ The data used to produce the plot from the literature along with the estimations are listed in Table S1. There is in remarkably good agreement with the predicted values of *p*_*min*_ for all the experimental studies using EHD printing in a wide range of feature sizes from hundreds of micrometers^20^ to 100 nm.^36^

### 3.3 Stable Printed Lines with Parallel Sides

**Figure 6:**
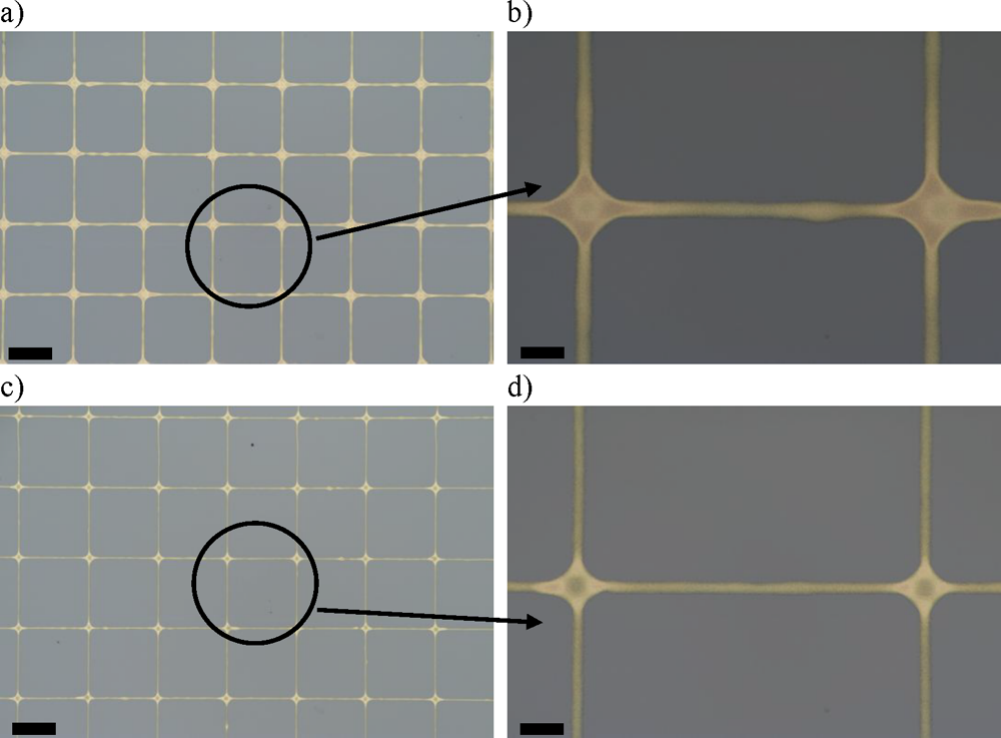
Printed silver grids on the Si substrate: a) and b) printed grids at a printing velocity of 2 mm s^–1^ and a drop spacing of 5 μm with a pulse width time of 0.5 ms and c) and d) printed grids at a printing velocity of 3 mm s^–1^ and a drop spacing of 3.75 μm with a pulse width time of 0.25 ms. The scale bar in a) and c) is 100 μm, and in b) and d) it is 20 μm.

The model predicts the minimum possible printable stable line width to be defined by the equilibrium diameter of an isolated sessile drop and is thus controlled by the printed drop volume and the contact angle. This assumes that the receding contact angle is zero (contact line pinning). To test this hypothesis, we printed two square grid arrays of Ag lines on the lower contact angle Si substrate, with a nominal drop volume of 11 and 4 fL using maximum drop spacing values as predicted by the model (Figure 6). Such fine Ag grids are suitable for touch screen and transparent electrode applications. The printing conditions and drop dimensions are displayed in Table 3. The line width measured in each case is close to the equilibrium diameter of an isolated drop, consistent with Stringer and Derby’s model.^29^ It is notable that in both sets of experiments there is a clear increase in the width of the printed lines at the nodes of the printed grids (Figure 6b and 6d). This is explained by fluid flow within the printed lines before ink solvent evaporation driven by the smaller surface curvature and lower Laplace pressure where the lines intersect at the nodes. However, this does not affect the line width between the nodes because of contact line pinning. There are occasional bulges visible in the lines between the printed nodes, but these are not related to the bulging instability at a small drop spacing but are likely to be associated with surface imperfections on the Si substrate or are drying defects that have been reported previously with the printing of lines using conventional inkjet printing.^30^ An important consequence of the fluid flow to the nodes in the printed grid arrays is that this limits the spacing of the lines.

**Table 3:** Printing and Drop Parameters for Square Grid Arrays Printed on Si Substrates with an Advancing Contact Angle of 11°

drop volume (fL)	printing speed (mm s^–1^)	drop spacing (μm)	line width (μm)	*d*_*eqm*_(μm)
11	2	5	8.2	8.3
4	3	3.75	5.7	5.9

### 3.4 Line Electrical Properties

**Figure 7:**
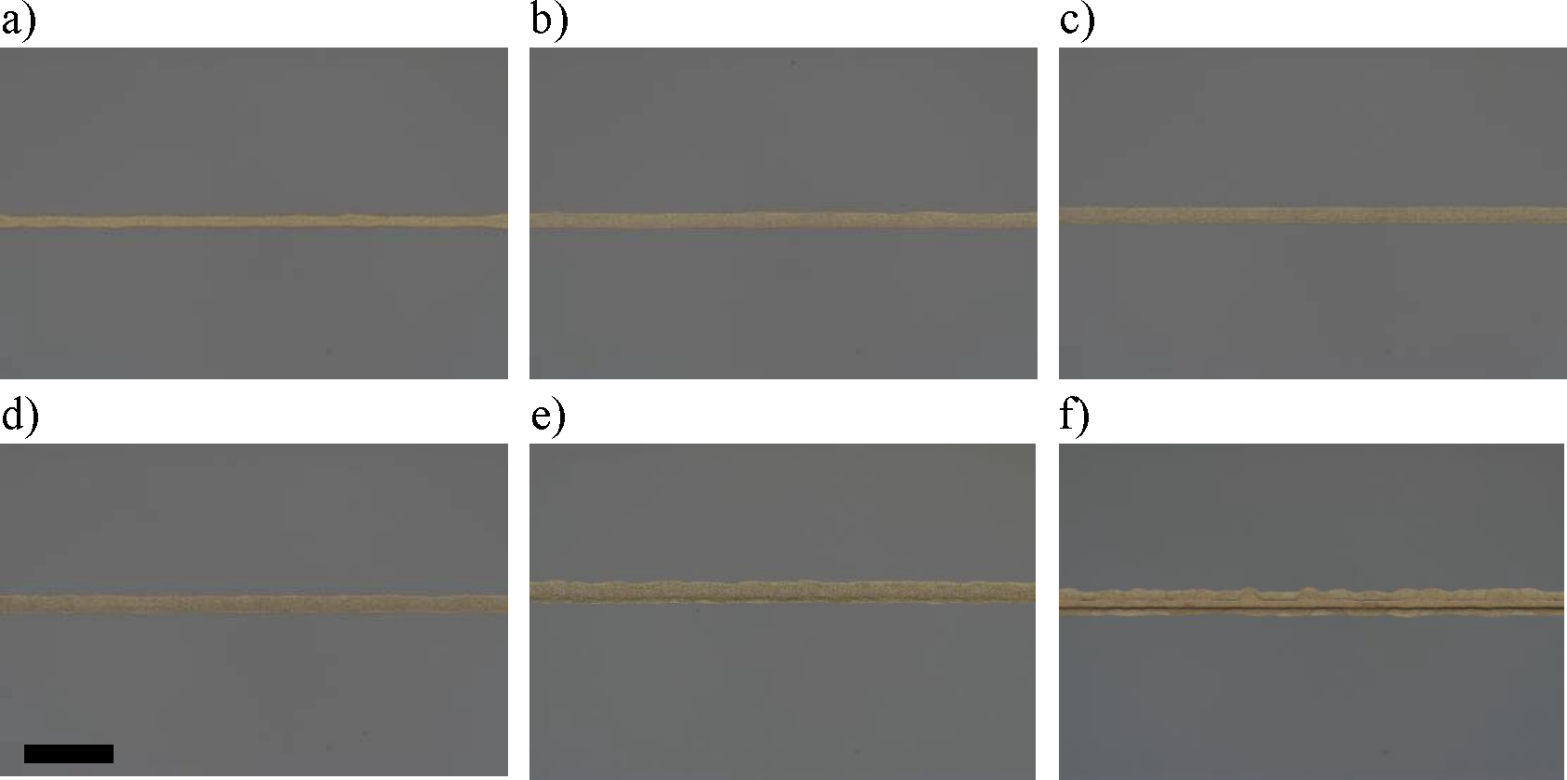
Optical images showing the morphologies of the printed lines used for electrical measurements after increasing the number of printed layers: a) 1 layer, b) 2 layers, c) 3 layers, d) 5 layers, e) 10 layers, and f) 15 layers. The scale bar is 50 μm.

**Figure 8:**
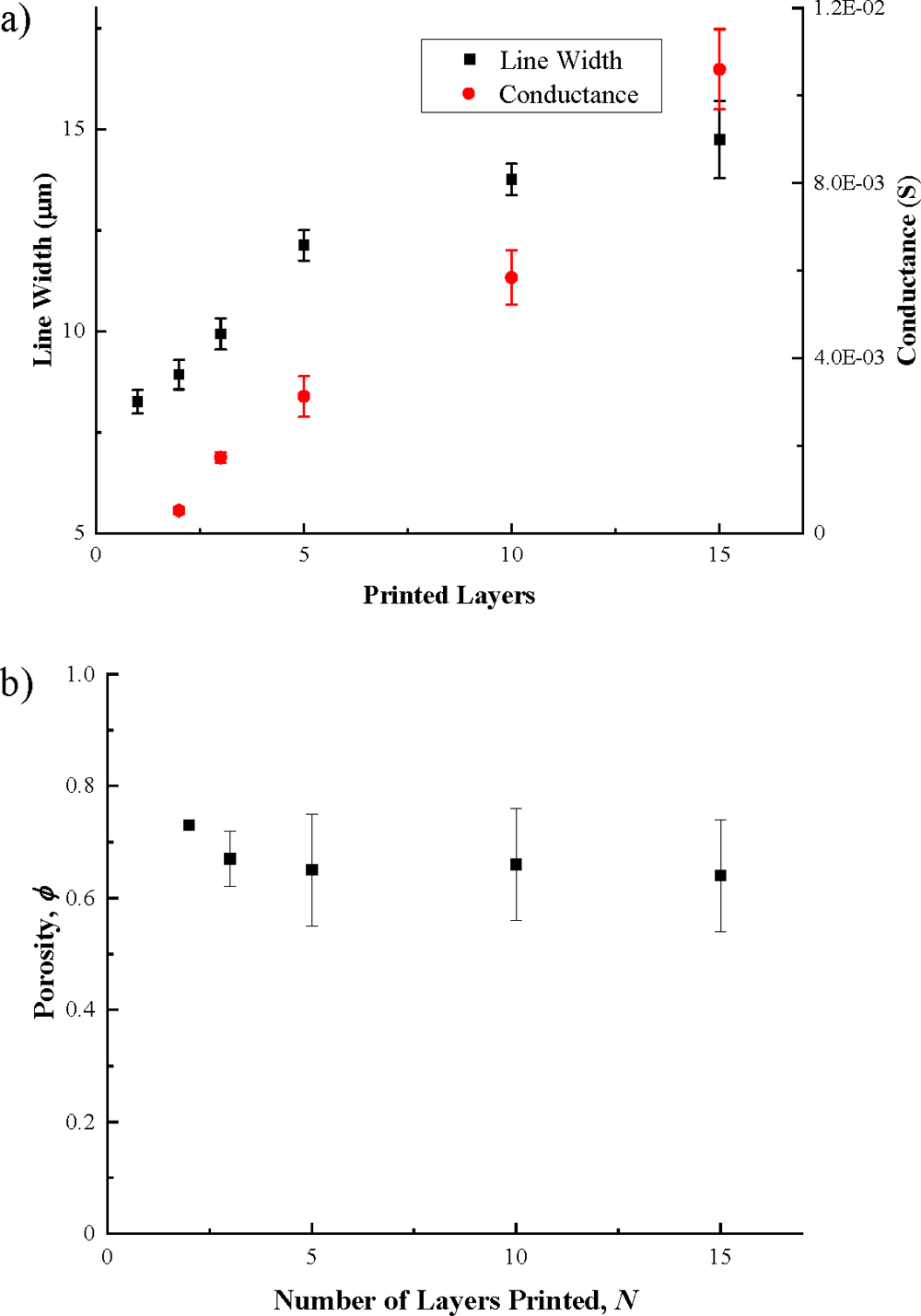
Printed line properties as a function of the number of printed layers on the Si substrate: a) mean line width and conductance and b) estimated porosity after sintering.

All structures printed on the Si substrate used the same printing conditions of applied voltage, pulse width time, and nozzle to substrate distance parameters, as used for the line stability study, with a printing speed of 2 mm s^–1^, a drop spacing of 4 μm, and a drop volume of 11 pL. The influence of total Ag volume in the printed lines was investigated by repeat printing the line with extra layers of ink up to a maximum of 15 layers. Optical images of the lines after heat treatment are shown in Figure 7. All the lines are stable with parallel sides, showing no bulges or ridge width deviation. However, there is some minor roughness to the lines that increases with increasing numbers of printed layers. Both the line width and the electrical conductance increase with the number of printed layers (Figure 8). The lines printed with a single layer had no measurable conductivity, but all lines with multiple printed layers were conducting. The properties of the lines printed with a single layer of overlapping drops illustrates a weakest link phenomenon, where the electrical properties critically depend on continuous drop overlap between many drops printed in sequence. Figure 4a shows that lines printed with an overlap of *p* = 5 μm are at the threshold of stable coalescence; thus, it is likely that the lines printed using a single line of drops are susceptible to single drop failure events within the 500 drops used to print the 2 mm line length.

Assuming the solvent is removed during annealing, the total deposited silver mass per unit length of the line printed on the substrate is
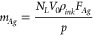
9where *N*_*L*_ is the number of printed layers, *ρ*_*ink*_ is the silver ink density (1450 kg m^–3^), and *F*_*Ag*_ is the silver mass fraction in the ink. Thermogravimetric analysis (TGA) determined *F*_*Ag*_ = 0.30 (Supporting Information Figure S7). Assuming that when a parallel sided line is observed, the lines have a uniform cross section and that after the ink has been heat treated, it contains a fraction φ of pores; the cross sectional area of the line is
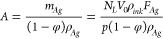
10where *ρ*_*Ag*_ is the density of silver. The conductance of the line, *G*, is determined by its length, *L*, cross sectional area, and conductivity, σ, of the sintered Ag ink:
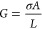
11

Bulk Ag has a conductivity of σ*_Ag_* = 6.21 × 10^7^ S m^–1^, and the conductivity of the sintered Ag is modified by its porosity and can be approximated as follows:^43^
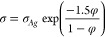
12Hence the conductance, *G*, of a printed line is
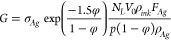
13The porosity of the sintered Ag can be estimated from the measured conductance values and combining eqs 12 and 13 (Figure 8b). The porosity of the line printed with two layers is >70%, but at greater line numbers, the porosity converges to around 65%, which is equivalent to an apparent conductivity in the range 10–20% of bulk Ag. This is a relatively large level of porosity; however, it is well-known that printed nanoparticle inks often show apparent conductivities significantly lower than bulk values. Reviews of the literature show that typical conductivity measurements for printed Ag range from 10%–50% of bulk silver, and this is consistent with the high levels of porosity found in our study.^44−47^

## 4 Conclusions

Models for the stability of lines produced by inkjet printing with drops in the pL to nL volume range are applicable to EHD printing with drops in the fL range. For a given drop size, stable regular lines can be printed only within a range of drop spacings, effectively limiting the range of line widths. The maximum drop spacing for regular line formation defines the minimum line width, which, in the case of a zero receding contact angle, is equivalent to the equilibrium diameter of a single ejected drop on the substrate. The minimum drop spacing, which defines the maximum stable line width, is defined by a dynamic equilibrium between the spreading of new drops arriving on the surface and a Laplace pressure driven flow along the existing liquid line. This equilibrium is determined by both the contact angle and the rate at which drops arrive at the surface (printing speed), with a higher printing speed increasing the range of drop spacing where stable lines are printable. The printing range is also extended if the ink contact angle decreases. The predictions of the model are also consistent with data on EHD printed lines with a range of substrates and inks published in the literature.

Guided by the predictions of the model, we are able to print Ag lines of width 5 μm using 4 fL drops and of width 8 μm using 11 fL drops. When printed in a grid pattern, fluid flow occurs to the intersections, but the zero receding contact angle ensures stable lines are retained at the minimum limiting line width. Lines printed with a single printing pass at the minimum line width do not show conductance over 2 mm lengths (approximately 500 drops); this indicates the susceptibility of printed lines to single drop failure events, e.g., local contamination or slight drop misplacement. Electrical conductance measurements show that the heat treated lines have properties consistent with a large level of internal porosity. However, the electrical properties are similar to those reported for the conductivity of inkjet printed lines produced using conventional inkjet printing of Ag nanoparticle inks.

## Supporting Information Available

Summary of rheological and printing data to produce Figure 5 from literature, full details of method used to determine conditions selected for EHD printing, complete table of EHD settings used for each set of printing conditions, complete set of images of printed lines under all experimental conditions used in study, and TGA results to confirm Ag content of ink (PDF)
