# Supplementary material for: Stability Bounds for Micron Scale Ag Conductor Lines Produced by Electrohydrodynamic Inkjet Printing
Source: ACS Appl Mater Interfaces. 2022 Aug 18;14(34):39601–9. doi: 10.1021/acsami.2c11133 (PMC9437868; doi:10.1021/acsami.2c11133)
Supplement: Supplementary file 1 — am2c11133_si_001.pdf [file am2c11133_si_001.pdf]

Supporting Information

Stability Bounds for Micron Scale Ag Conductor Lines Produced by Electrohydrodynamic Inkjet Printing

*Jinxin Yang, Pei He, and Brian Derby**

J. Yang, B. Derby

Department of Materials, University of Manchester, Oxford Rd., Manchester, M13 9PL, UK

E-mail: brian.derby@manchester.ac.uk

P. He

School of Physics and Electronics, Hunan Key Laboratory of Nanophotonics and Devices, Central South University, Changsha, Hunan 410083, P. R. China

EHD DOD Printed Lines in Previous Publications

Table S1 Summary of the rheological and printing data to produce Figure 5 from the literature. The data with "*" means that the data is not provided by the literature and thus estimated reasonably. The yellow data means that the lines are unstable with bulges.

Ink Material	Surface Tension (mN m^{-1})	Viscosity (mPa·s)	Contact Angle (degree)	Deposited Droplet Diameter (µm)	Printing Speed (mm s^{-1})	Drop Spacing (µm)	Line Width (µm)	Ref.
Ag Nps	30	15.0*	12.0*	335.5	100.0	100.0	346.8	[S1]
Ag Nps	24.1	13.0	35.0*	17.0*	5.0	10.0*	15.5	[S2]
Ag Nps	24.1	13.0	35.0*	14.5*	7.5	7.5*	15.6	[S2]
Ag Nps	24.1	13.0	35.0*	15.9*	5.0	6.6*	18.2	[S2]
Ag Nps	24.1	13.0	35.0*	14.5*	5.0	5.0*	20.0	[S2]
Ag Nps	31.0	14.4	50.0	43.0	15.0	18.8	57.0	[S3]
Ag Nps	31.0	14.4	35.0	53.0	20.0	25.0	60.0	[S3]
Ag Nps	31.0	14.4	50.0	43.0	10.0	12.5	60.0	[S3]
Ag Nps	31.0	14.4	50.0	43.0	20.0	25.0	43.9	[S3]
Ag Nps	31.0	14.4	35.0	53.0	15.0	18.8	63.0	[S3]
Ag Nps	31.0	14.4	35.0	53.0	10.0	12.5	72.0	[S3]
Ag Nps	31.0	14.4	50.0	43.0	5.0	6.3	90.0	[S3]
Ag Nps	31.0	14.4	35.0	53.0	5.0	6.3	118.0	[S3]
Ag Nps	24.0	9.0	12.0*	7.1*	50.0	2.5*	9.2	[S4]
Ag Nps	24.0	9.0	12.0*	9.0*	100.0	5.0*	9.3	[S4]
Ag Nps	24.0	9.0	12.0*	7.4*	50.0	2.5*	9.5	[S4]
Ag Nps	24.0	9.0	12.0*	9.8*	100.0	5.0*	10.5	[S4]
Ag Nps	24.0	9.0	12.0*	8.3*	50.0	2.5*	11.6	[S4]
Ag Nps	24.0	9.0	12.0*	11.0*	100.0	5.0*	12.5	[S4]
Ag Nps	24.0	9.0	12.0*	7.1*	25.0	1.3*	13.0	[S4]
Ag Nps	24.0	9.0	12.0*	9.0*	50.0	2.5*	13.1	[S4]
Ag Nps	24.0	9.0	12.0*	7.4*	25.0	1.3*	13.8	[S4]
Ag Nps	24.0	9.0	12.0*	9.8*	50.0	2.5*	14.9	[S4]
Ag Nps	24.0	9.0	12.0*	8.3*	25.0	1.3*	16.4	[S4]
Ag Nps	24.0	9.0	12.0*	11.0*	50.0	2.5*	17.7	[S4]
Ag Nps	24.0	9.0	12.0*	9.0*	25.0	1.3*	18.5	[S4]
Ag Nps	24.0	9.0	12.0*	9.8*	25.0	1.3*	21.1	[S4]
Ag Nps	24.0	9.0	12.0*	11.0*	25.0	1.3*	25.1	[S4]
Ag Nps	24.0	8.0	50.0*	65.0*	2.0	25*	81.6	[S5]
Ag Nps	24.0	8.0	50.0*	85.0*	2.0	50*	86.3	[S5]
Ag Nps	30.0	12.0	12.0*	50	0.4	20	60.7	[S6]
Ag NP	36.0	15.0	35.0*	3.5	8.5	1.4	4.0	[S7]
Ag Nps	36.0	15.0	35.0*	3.5	7.5	1.3	4.5	[S7]
Ag Nps	36.0	15.0	35.0*	4.2	10.0	2.0	4.9	[S8]
Ag Nps	36.0	15.0	35.0*	4.2	7.0	1.4	5.3	[S8]
Au Nps	26.6*	2.1*	10.0	0.1*	0.18	0.045*	0.12	[S9]
Cu Nps	36.9	7.6	23.0*	23.5*	20.0	10.0*	27.8	[S10]
Cu Nps	36.9	7.6	23.0*	24.8*	20.0	10.0*	30.0	[S10]
Cu Nps	36.9	7.6	23.0*	26.0*	20.0	10.0*	32.2	[S10]
Cu Nps	41.4	4.5	35.0*	10.7*	25.0	5.0*	12.0	[S11]
Cu Nps	41.4	4.5	35.0*	10.9*	25.0	5.0*	12.5	[S11]
ITO Nps	40.0*	10.0*	23.0	48.3*	50.0	25.0*	58.0	[S12]
ITO Nps	40.0*	10.0*	23.0	48.3*	20.0	10.0*	65.0	[S12]

**ITO Nps**	40.0*	10.0*	23.0	48.3*	10.0	5.0*	119.0	[S12]
**ITO Nps**	40.0*	10.0*	23.0	48.3*	5.0	2.5*	167.0	[S12]
**ITO Nps**	40.0*	10.0*	23.0	48.3*	1.0	0.5*	230.0	[S12]
**Graphene**	30.0	12.0	12.0*	35.0*	1.0	10.0	50.0	[S13]
**Graphene**	30.0	12.0	12.0*	72.5*	1.0	10.0	150.0	[S13]
**Polymer**	30.0*	18.0*	10.0*	0.67*	0.03	0.4	0.75	[S14]
**Polymer**	30.0*	18.0*	10.0*	0.67*	0.02	0.3	0.8	[S14]
**Polymer**	30.0*	18.0*	10.0*	0.67*	0.01	0.1	1.0	[S14]
**Polymer**	30.0*	18.0*	10.0*	0.67*	0.008	0.09	1.3	[S14]
**Polymer**	30.0*	18.0*	10.0*	0.67*	0.005	0.06	1.7	[S14]

Actuation Conditions used to Generate Droplets

The threshold voltage, V_{thr}, for drop ejection at a specific height, H, above the Z10 substrate, was obtained by increasing the pulse amplitude voltage from a bias voltage of 100 V to the voltage at which droplet ejection occurs. The threshold voltage for droplet ejection increases with increased nozzle to substrate distance (Figure S1a). The electric field strength at the nozzle tip can be estimated by using a model of a semi-infinite wire perpendicular to an infinite planar counter electrode:[S15, S16]

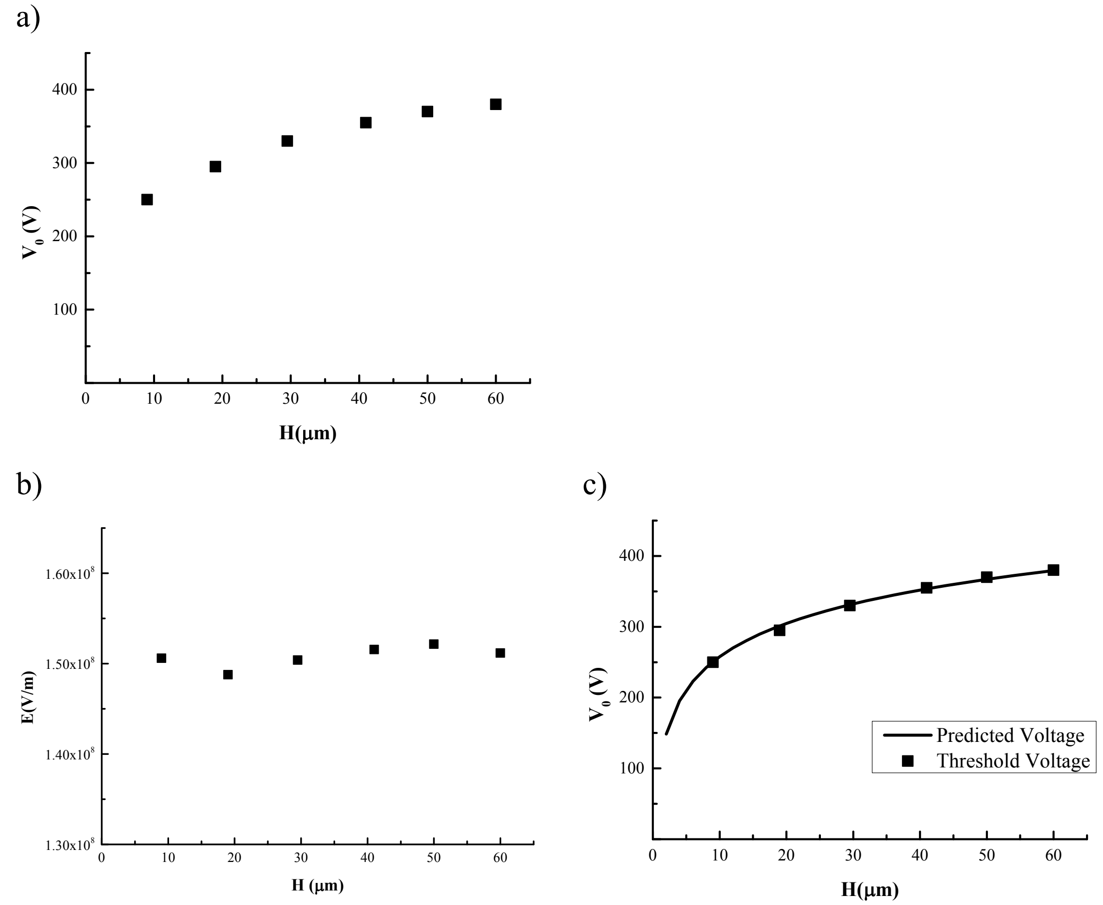

Figure S1. Threshold voltage (V_o) for ejection and nozzle tip electric field (E) as a function of nozzle to substrate distance (H) on the UV-Ozone treated silicon: a) the relationship between V_o and H ; b) the relationship between E and H; c) predicted V_o as a function of H by using average E and Equation S1.

$$E = \frac{4V_0}{d_N ln\,(\frac{8H}{d_N})} \qquad \text{(S1)}$$

where d_N is the inner diameter of the nozzle, which is around 1.8 μm, and V_0 is the applied voltage. The electric field strength, calculated using Equation S1, for the threshold jetting

voltage at different nozzle to substrate distance is plotted in **Figure** b. The threshold electric field strength for jetting is approximately constant over all nozzle to substance distances studied. This is consistent with the predictions of the relationship between fluid flow rate, Q, and EHD jetting parameters proposed by Chen *et al*:[S17]

$$Q \approx \frac{\pi d_N^4}{128\mu L}\left(\frac{1}{2}\varepsilon_0 E^2 + \Delta P - \frac{2\gamma}{d_N}\right) \quad \text{(S2)}$$

where L is the length of the nozzle, μ is the fluid viscosity, ε_0 is the vacuum permittivity, E is the strength of applied electric field, ΔP is the applied hydrostatic pressure, and γ is the surface tension of the fluid. The threshold for jetting requires the sum of the electric pressure $\varepsilon_0 E^2/2$ and hydrostatic pressure ΔP to exceed the capillary pressure, $2\gamma/d_N$, at the nozzle. At the threshold for jetting, the flow rate Q should be constant. Given that the hydrostatic pressure and capillary pressure remain constant at the threshold for jetting, independent of nozzle to substrate distance conditions, the electric pressure also remains the same, thus explaining the constant electric field strength at the threshold for droplet ejection. This agreement between the predicted electric field strength and observation, indicates that our printing conditions can be described by EHD principles and meniscus behaviour, although it is difficult to observe the meniscus deformation during printing in our system due to the relatively small nozzle size.

By using the average electric field strength from the calculated results, the predicted threshold voltage for jetting can be obtained as a function of nozzle to substrate distance by using Equation S1. This is shown in Figure S2c, with the predicted curve agreeing well with our measured results. After a series of experiments, we chose an applied voltage pulse of 310 V with bias voltage 100 V, with a nozzle to substrate distance of 20 μm, for optimum printing conditions. The pulse voltage is chosen to be just above the threshold voltage for jetting (295V) to achieve stable minimised feature size, and the nozzle to substrate distance is chosen with an intermediate value to minimise the deviation in the printed feature size at small values and unstable spraying at large distances.

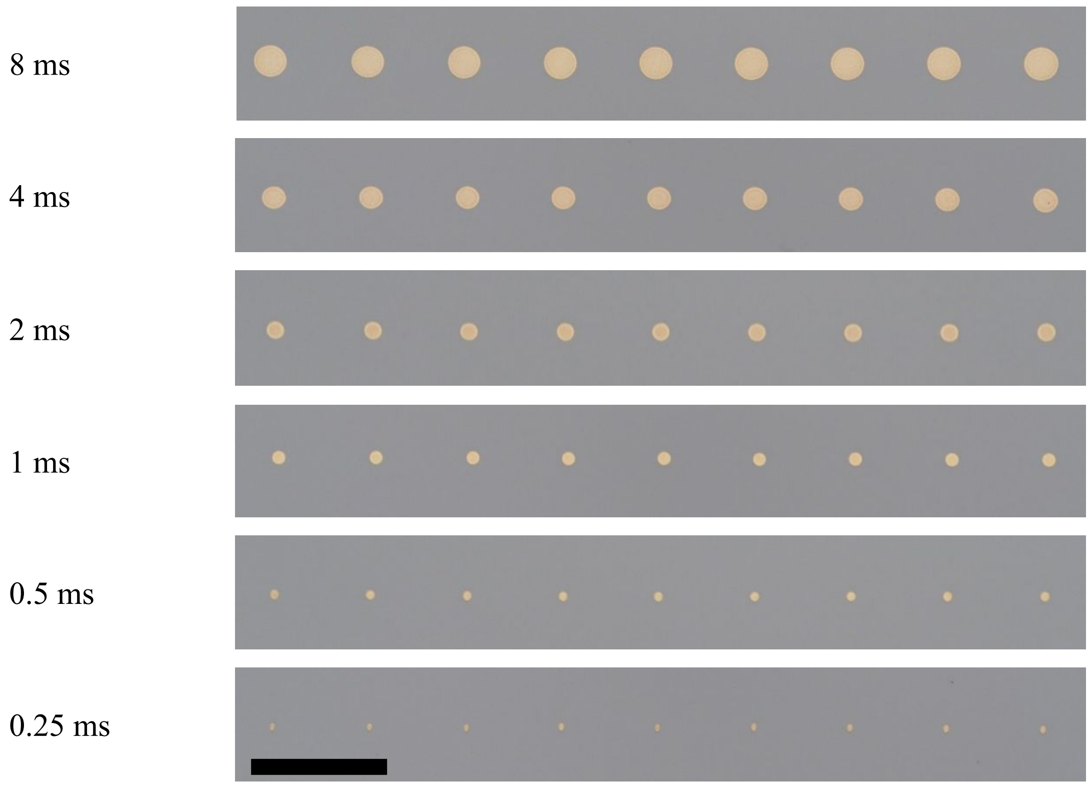

Figure S2 Single dots deposited on 10 min UV-Ozone treated silicon as a function of pulse width time at an applied voltage of 310 V. The nozzle to substrate distance is 20 μm. The scale bar is 100 μm.

For pulsed EHD inkjet printing, the total ejected volume per pulse is determined by the ejected flow rate from the cone and the pulse width time. This can be calculated as:

$$V = Q \cdot T_p = Q\frac{x_p}{f} \tag{S3}$$

where V is the total ejected drop volume per pulse, Q is the jetting flow rate, T_p is the pulse width time, x_p is the duty ratio of the pulse, and f is the applied pulse frequency. To investigate the relationship between deposited droplet diameter and pulse width time, a series of individual dots were printed onto 10 min UV-Ozone treated silicon at different pulse width times, as shown in Figure S2. It can be seen that stable, uniform dots were obtained over a range of pulse width times, with the dot diameter increasing dramatically at elevated pulse width time. Assuming the jetting flow rate remains constant during printing, as the electric pressure, hydrostatic pressure, and capillary pressure remain the same in our experiment set up, the deposited droplet diameter is proportional to the cube root of the pulse width time:

$$d_{eqm} = \beta_{eqm}\sqrt[3]{\frac{6Q}{\pi}t} \tag{S4}$$

where β_{eqm} is the ratio of the diameter of a sessile droplet to the diameter of the equivalent volume spherical drop and controlled by the contact angle, θ, with

$$\beta_{eqm} = \sqrt[3]{\frac{8}{tan\frac{\theta}{2}(3 + tan^2\frac{\theta}{2})}} \quad \text{(S5)}$$

The flow rate, Q, in Equation 4 was obtained by using the mean deposited dot diameter at pulse width time 0.5 ms, and the calculated value is 2.39×10^{-14} m^3s^{-1}. By using the calculated flow rate and the above equation, the dot diameter as a function of pulse width time is predicted, as shown in Figure S3, confirming the assumption that the flow rate remains constant as the pulse width is varied.

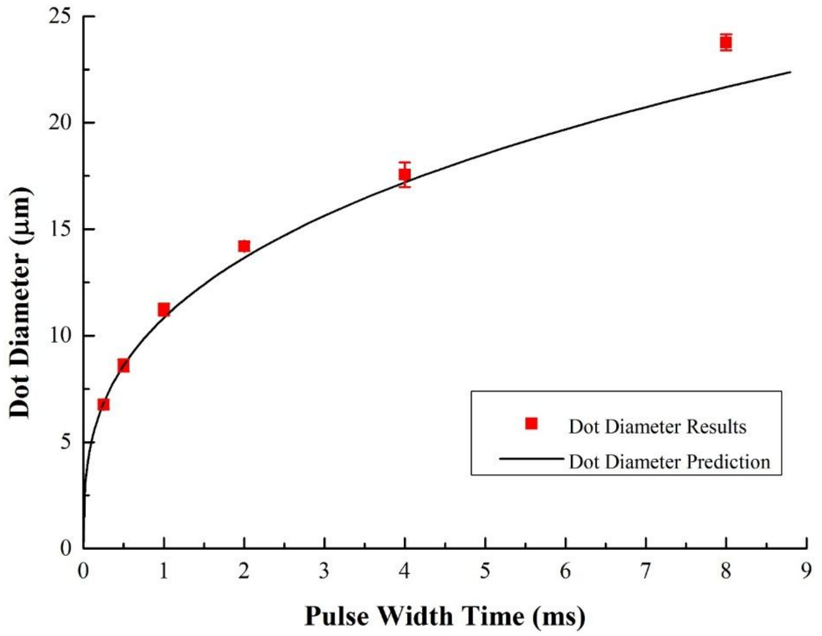

Figure S3 The deposited dot diameter on the UV-Ozone treated silicon substrate as a function of pulse width time, along with the dot diameter prediction curve.

For our experiments, it is necessary to provide constant drop volume at a range of drop spacings and transverse printing velocities. However, the drop generation frequency is a function of drop spacing and velocity. Thus, to achieve a constant ejected volume per pulse, the pulse width time T_p should remain constant as the frequency is changed, requiring the applied duty ratio of pulse x_p to also change with frequency, in accordance with Equation S3. The appropriate duty ratio and frequency for the printing velocity and drop spacing conditions used in the study are listed in Table S1 – Table S5.

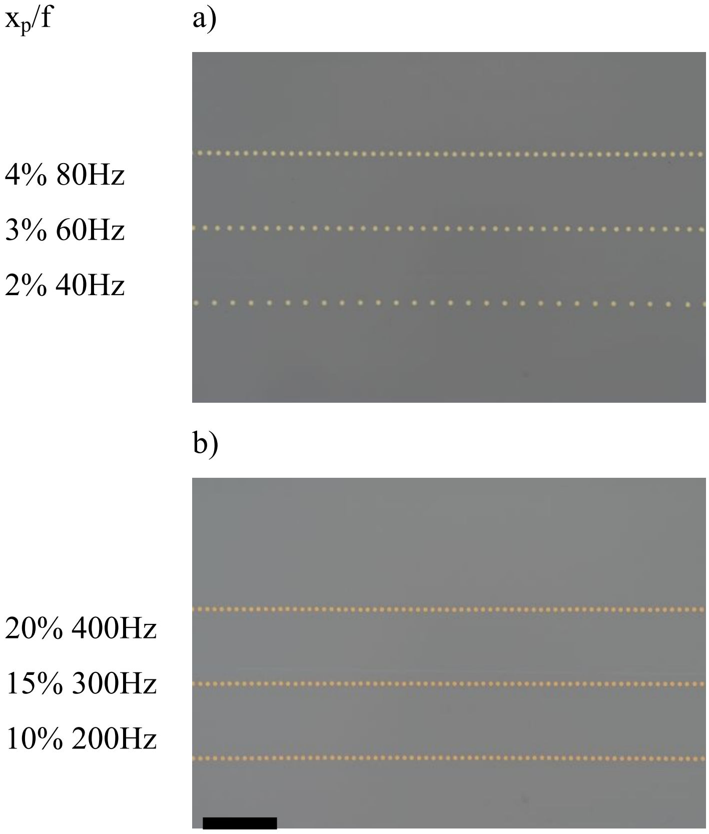

Figure S4 Printed dots at applied voltage 310 V, nozzle to substrate distance 20 µm, and pulse width time 0.5 ms, with different jetting frequency and duty ratio: a) at low frequency and fixed print velocity of 1 mms^{-1}, b) at high frequency and fixed drop spacing of 10 µm. The scale bar is 100 µm.

To confirm the deposited droplet size at various printing frequencies, printing was carried out onto 10 min UV-Ozone treated silicon at a drop spacing significantly larger than the deposited droplet diameter, and with a fixed pulse width time, 0.5 ms. As shown in Figure S4, a series of stable circular dots were obtained at various printing frequency conditions. The average deposited droplet diameter is 8.4 µm with a standard deviation of 0.3 µm, indicating that droplets with identical size can be obtained at various jetting frequencies. The ejected volume per pulse can be calculated from the deposited droplet diameter and the contact angle on the substrate by using volume conservation, assuming a spherical cap geometry:

$$V_0 = \frac{\pi tan\frac{\theta}{2}(3 + tan^2\frac{\theta}{2})}{48} d_{eqm}^3 \qquad \text{(S6)}$$

where θ is the contact angle of the deposited droplet on the substrate, and d_{eqm} is the deposited droplet diameter. Here we used the advancing contact angle (θ_a) instead of the equilibrium contact angle (θ_{eqm}), as the droplet spreads and advances on the solid surface

after impact. By substituting the average deposited droplet diameter of 8.4 μm and the advancing contact angle of 10.9° into Equation 6, the estimated drop volume per pulse from our printing setup is around 11 fL, which is 2 to 3 orders of magnitude smaller than that generated from a commercial piezoelectric inkjet printer.

Single Pulse Waveform used with Z10 Substrates

Table S2 Printing parameters at the printing speed of 2 mms^{-1}.

V (mm/s)	P (μm)	F (Hz)	Pulse Time (ms)	Waveform (%)
1.96	7	280	0.5	14
1.92	6	320	0.5	16
2	5	400	0.5	20
2	4	500	0.5	25
2	3.3	600	0.5	30
2	2.5	800	0.5	40
2	2	1000	0.5	50

Table S3 Printing parameters at the printing speed of 1 mms^{-1}.

V (mm/s)	P (μm)	F (Hz)	Pulse Time (ms)	Waveform (%)
0.98	7	140	0.5	7
0.96	6	160	0.5	8
1	5	200	0.5	10
0.96	4	240	0.5	12
1	3.3	300	0.5	15
1	2.5	400	0.5	20
1	2	500	0.5	25
1	1	1000	0.5	50

Table S4 Printing parameters at the printing speed of 0.4 mms^{-1}.

V (mm/s)	P (µm)	F (Hz)	Pulse Time (ms)	Waveform (%)
0.42	7	60	0.5	3
0.36	6	60	0.5	3
0.4	5	80	0.5	4
0.4	4	100	0.5	5
0.4	3.3	120	0.5	6
0.4	2.5	160	0.5	8
0.4	2	200	0.5	10
0.4	1	400	0.5	20

Table S5 Printing parameters at the printing speed of 0.2 mms^{-1}.

V (mm/s)	P (µm)	F (Hz)	Pulse Time (ms)	Waveform (%)
0.28	7	40	0.5	2
0.24	6	40	0.5	2
0.2	5	40	0.5	2
0.24	4	60	0.5	3
0.2	3.3	60	0.5	3
0.2	2.5	80	0.5	4
0.2	2	100	0.5	5
0.2	1	200	0.5	10

Table S6 Printing parameters at the printing speed of 0.1 mms^{-1}.

V (mm/s)	P (µm)	F (Hz)	Pulse Time (ms)	Waveform (%)
0.14	7	20	0.5	1
0.12	6	20	0.5	1
0.1	5	20	0.5	1
0.08	4	20	0.5	1
0.132	3.3	40	0.5	2
0.1	2.5	40	0.5	2
0.12	2	60	0.5	3
0.08	1	80	0.5	4

Bipolar Waveform used with Z40 Substrates

Table S7 Printing parameters at the printing speed of 2 mms^{-1}.

V (mm/s)	P (μm)	F (Hz)	Pulse Time (ms)	Waveform (%)
1.96	7	140	0.5	7
1.92	6	160	0.5	8
2	5	200	0.5	10
1.92	4	240	0.5	12
2	3.3	300	0.5	15
2	2.5	400	0.5	20
2	2	500	0.5	25
2	1	1000	0.5	50

Table S8 Printing parameters at the printing speed of 0.4 mms^{-1}.

V (mm/s)	P (μm)	F (Hz)	Pulse Time (ms)	Waveform (%)
0.56	7	40	0.5	2
0.48	6	40	0.5	2
0.4	5	40	0.5	2
0.48	4	60	0.5	3
0.4	3.3	60	0.5	3
0.4	2.5	80	0.5	4
0.4	2	100	0.5	5
0.4	1	200	0.5	10

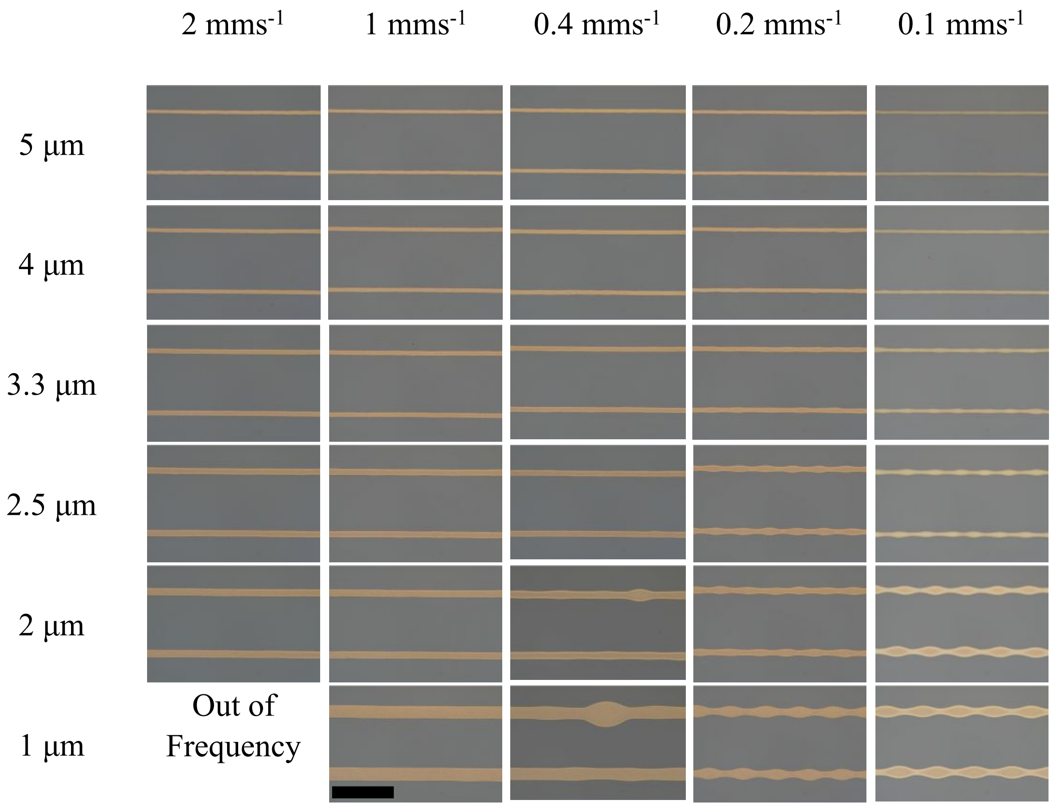

Figure S5 Lines printed on the Z10 substrate with printing speed in the range 0.1 – 2 mms^{-1} and drop spacing 1 – 5 µm, with 11 fL drops. The scale bar is 100 µm.

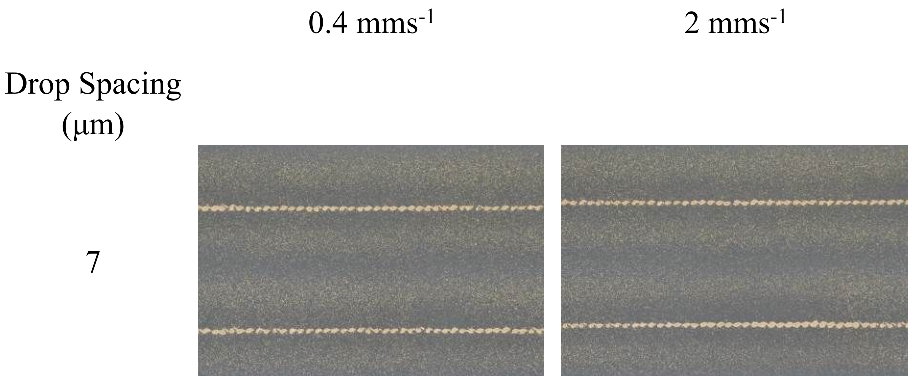

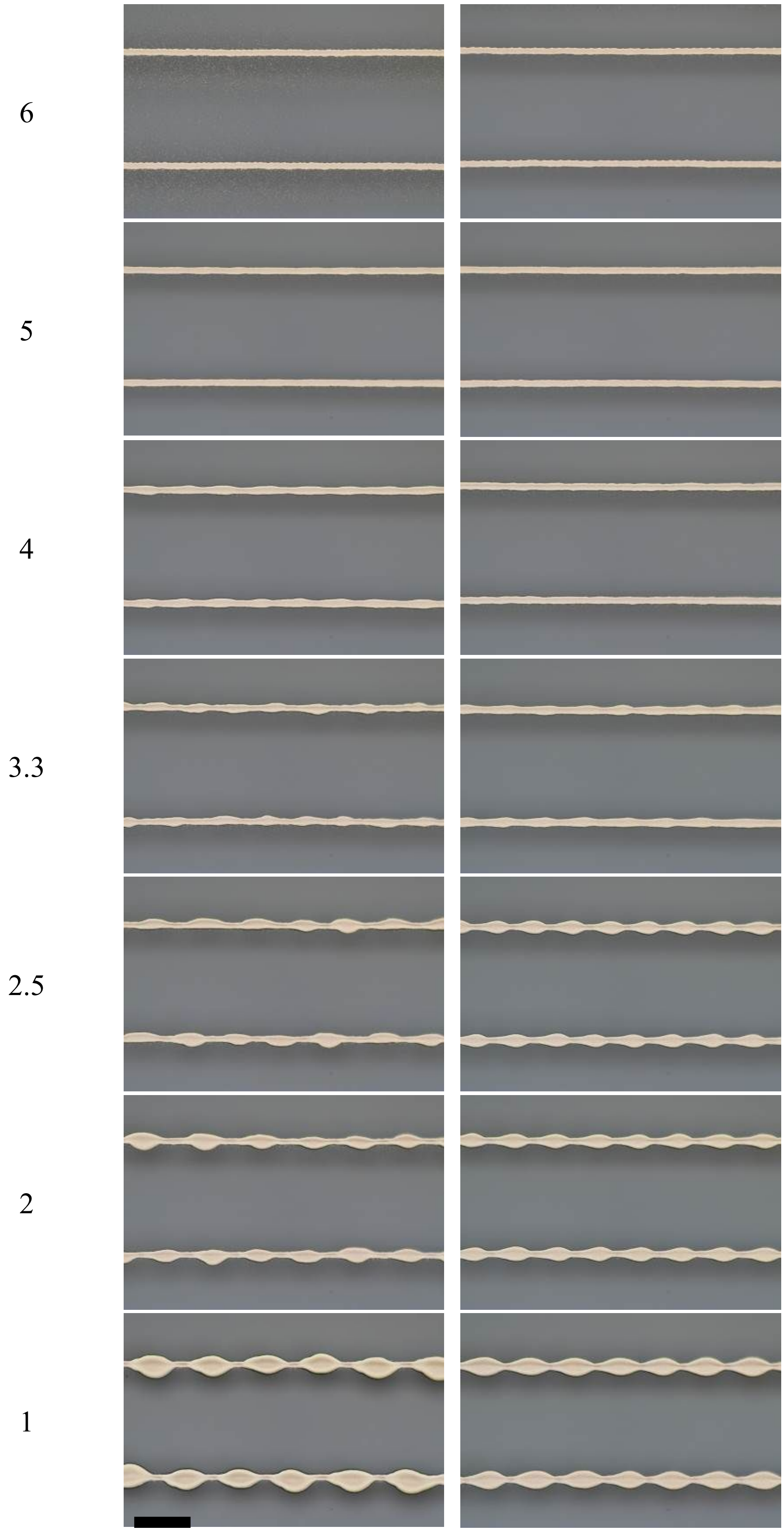

Figure S6 Lines printed on the Z40 substrate, with drop spacing ranged from 7 to 1 μm, and printing speed of 2 and 0.4 mms^{-1}. The scale bar is 50 μm.

The contact angle data with “*” are estimated from the rheological properties of the applied ink and the substrates applied. The deposited droplet diameter data and drop spacing data are estimated from the line width given by the literature using Equation 2 and 3a.

Thermogravimetric Analysis of the Heat Treatment of the Ag Ink

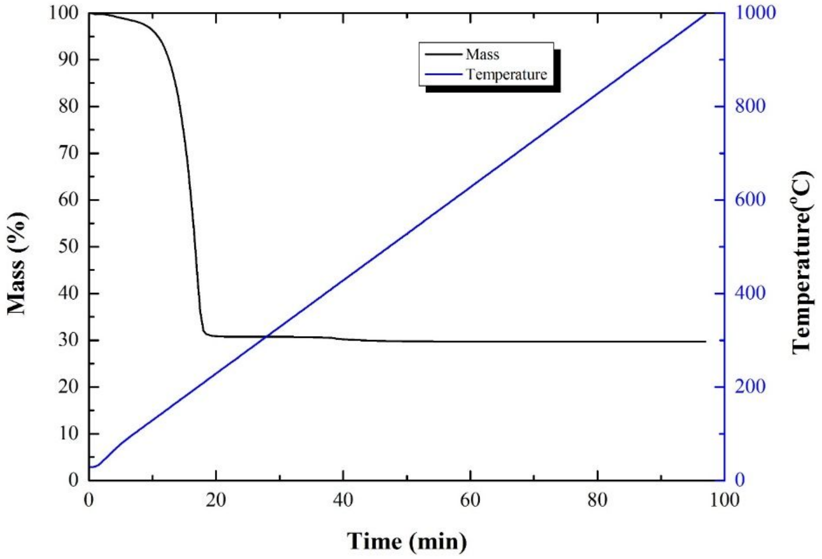

Figure S7 TGA test of the silver nanoparticle ink.

Supporting Information Reference

[S1] Choi, J.; Kim, Y.-J.; Lee, S.; Son, S. U.; Ko, H. S.; Nguyen, V. D.; Byun, D. Drop-on-demand printing of conductive ink by electrostatic field induced inkjet head. *Appl. Phys. Lett.* **2008,** *93*, 193508.

[S2] Phung, T. H.; Kim, S.; Kwon, K.-S. A high speed electrohydrodynamic (EHD) jet printing method for line printing. *J Micromech Microeng* **2017,** *27*, 095003.

[S3] Park, J.; Kim, B.; Kim, S.-Y.; Hwang, J. Prediction of drop-on-demand (DOD) pattern size in pulse voltage-applied electrohydrodynamic (EHD) jet printing of Ag colloid ink. *Appl. Phys. A* **2014,** *117*, 2225-2234.

[S4] Son, S.; Lee, S.; Choi, J. Fine metal line patterning on hydrophilic non-conductive substrates based on electrohydrodynamic printing and laser sintering. *J Electrostat* **2014,** *72*, 70-75.

[S5] Kim, Y.; Jang, S.; Oh, J. H. High-resolution electrohydrodynamic printing of silver nanoparticle ink via commercial hypodermic needles. *Appl. Phys. Lett.* **2015,** *106*, 014103.

[S6] Wang, X.; Xu, L.; Zheng, G.; Cheng, W.; Sun, D. Pulsed electrohydrodynamic printing of conductive silver patterns on demand. *Sci. China Technol. Sci.* **2012,** *55*, 1603-1607.

[S7] Qin, H.; Dong, J.; Lee, Y.-S. AC-pulse modulated electrohydrodynamic jet printing and electroless copper deposition for conductive microscale patterning on flexible insulating substrates. *Robot Comput Integr Manuf* **2017,** *43*, 179-187.

[S8] Wei, C.; Qin, H.; Chiu, C.-P.; Lee, Y.-S.; Dong, J. Drop-on-demand E-jet printing of continuous interconnects with AC-pulse modulation on highly insulating substrates. *J Manuf Syst* **2015,** *37*, 505-510.

[S9] Galliker, P.; Schneider, J.; Eghlidi, H.; Kress, S.; Sandoghdar, V.; Poulikakos, D. Direct printing of nanostructures by electrostatic autofocussing of ink nanodroplets. *Nat. Commun.* **2012,** *3*, 890.

[S10] Khan, A.; Rahman, K.; Kim, D. S.; Choi, K. H. Direct printing of copper conductive micro-tracks by multi-nozzle electrohydrodynamic inkjet printing process. *J. Mater. Process. Technol.* **2012,** *212*, 700-706.

[S11] Rahman, K.; Khan, A.; Muhammad, N. M.; Jo, J.; Choi, K.-H. Fine-resolution patterning of copper nanoparticles through electrohydrodynamic jet printing. *J Micromech Microeng* **2012,** *22*, 065012.

[S12] Li, X. L.; Jung, E. M.; Kim, K. S.; Oh, J. H.; An, T. K.; Lee, S. W.; Kim, S. H. Printed Water-Based ITO Nanoparticle via Electrohydrodynamic (EHD) Jet Printing and Its Application of ZnO Transistors. *Electron. Mater. Lett.* **2019,** *15*, 595-604.

[S13] Zhao, K.; Wang, D.; Li, K.; Jiang, C.; Wei, Y.; Qian, J.; Feng, L.; Du, Z.; Xu, Z.; Liang, J. Drop-on-Demand Electrohydrodynamic Jet Printing of Graphene and Its Composite Microelectrode for High Performance Electrochemical Sensing. *J. Electrochem. Soc.* **2020,** *167*, 107508.

[S14] Onses, M. S.; Ramírez-Hernández, A.; Hur, S.-M.; Sutanto, E.; Williamson, L.; Alleyne, A. G.; Nealey, P. F.; de Pablo, J. J.; Rogers, J. A. Block Copolymer Assembly on Nanoscale Patterns of Polymer Brushes Formed by Electrohydrodynamic Jet Printing. *ACS Nano* **2014,** *8*, 6606-6613.

[S15] Marginean, I.; Nemes, P.; Vertes, A. Order-chaos-order transitions in electrosprays: The electrified dripping faucet. *Phys. Rev. Lett.* **2006,** *97*, 064502.

[S16] Choi, H. K.; Park, J.-U.; Park, O. O.; Ferreira, P. M.; Georgiadis, J. G.; Rogers, J. A. Scaling laws for jet pulsations associated with high-resolution electrohydrodynamic printing. *Appl. Phys. Lett.* **2008,** *92*, 123109.

[S17] Chen, C.-H.; Saville, D.; Aksay, I. A. Scaling laws for pulsed electrohydrodynamic drop formation. *Appl. Phys. Lett.* **2006,** *89*, 124103.
